# Supplementary material for: OpenMM 7: Rapid development of high performance algorithms for molecular dynamics
Source: PLoS Comput Biol. 2017 Jul 26;13(7):e1005659. doi: 10.1371/journal.pcbi.1005659 (PMC5549999; doi:10.1371/journal.pcbi.1005659)
Supplement: S1 Source Code — (ZIP) [file pcbi.1005659.s002.zip › OpenMM-7.0.1-Source/docs/api-python/generated/simtk.openmm.openmm.CustomManyParticleForce.html]

CustomManyParticleForce — OpenMM 7.0.1.dev-5e86c4f documentation


# CustomManyParticleForce¶

*class* `simtk.openmm.openmm.``CustomManyParticleForce`(*\*args*)¶
:   This class supports a wide variety of nonbonded N-particle interactions, where N is user specified. The interaction energy is determined by an arbitrary, user specified algebraic expression that is evaluated for every possible set of N particles in the system. It may depend on the positions of the individual particles, the distances between pairs of particles, the angles formed by sets of three particles, and the dihedral angles formed by sets of four particles.

    Be aware that the cost of evaluating an N-particle interaction increases very rapidly with N. Values larger than N=3 are rarely used.

    We refer to a set of particles for which the energy is being evaluated as p1, p2, p3, etc. The energy expression may depend on the following variables and functions:

    > - x1, y1, z1, x2, y2, z2, etc.: The x, y, and z coordinates of the particle positions. For example, x1 is the x coordinate of particle p1, and y3 is the y coordinate of particle p3.
    > - distance(p1, p2): the distance between particles p1 and p2 (where “p1” and “p2” may be replaced by the names of whichever particles you want to calculate the distance between).
    > - angle(p1, p2, p3): the angle formed by the three specified particles.
    > - dihedral(p1, p2, p3, p4): the dihedral angle formed by the four specified particles.
    > - arbitrary global and per-particle parameters that you define.

    To use this class, create a CustomManyParticleForce object, passing an algebraic expression to the constructor that defines the interaction energy of each set of particles. Then call addPerParticleParameter() to define per-particle parameters, and addGlobalParameter() to define global parameters. The values of per-particle parameters are specified as part of the system definition, while values of global parameters may be modified during a simulation by calling Context::setParameter().

    Next, call addParticle() once for each particle in the System to set the values of its per-particle parameters. The number of particles for which you set parameters must be exactly equal to the number of particles in the System, or else an exception will be thrown when you try to create a Context. After a particle has been added, you can modify its parameters by calling setParticleParameters(). This will have no effect on Contexts that already exist unless you call updateParametersInContext().

    Multi-particle interactions can be very expensive to evaluate, so they are usually used with a cutoff distance. The exact interpretation of the cutoff depends on the permutation mode, as discussed below.

    CustomManyParticleForce also lets you specify “exclusions”, particular pairs of particles whose interactions should be omitted from force and energy calculations. This is most often used for particles that are bonded to each other. If you specify a pair of particles as an exclusion, \_all\_ sets that include those two particles will be omitted.

    As an example, the following code creates a CustomManyParticleForce that implements an Axilrod-Teller potential. This is an interaction between three particles that depends on all three distances and angles formed by the particles.

    ```
    CustomManyParticleForce* force = new CustomManyParticleForce(3,
        "C*(1+3*cos(theta1)*cos(theta2)*cos(theta3))/(r12*r13*r23)^3;"
        "theta1=angle(p1,p2,p3); theta2=angle(p2,p3,p1); theta3=angle(p3,p1,p2);"
        "r12=distance(p1,p2); r13=distance(p1,p3); r23=distance(p2,p3)");
    force->setPermutationMode(CustomManyParticleForce::SinglePermutation);
    ```

    This force depends on one parameter, C. The following code defines it as a global parameter:

    ```
    force->addGlobalParameter("C", 1.0);
    ```

    Notice that the expression is symmetric with respect to the particles. It only depends on the products cos(theta1)\*cos(theta2)\*cos(theta3) and r12\*r13\*r23, both of which are unchanged if the labels p1, p2, and p3 are permuted. This is required because we specified SinglePermutation as the permutation mode. (This is the default, so we did not really need to set it, but doing so makes the example clearer.) In this mode, the expression is only evaluated once for each set of particles. No guarantee is made about which particle will be identified as p1, p2, etc. Therefore, the energy \_must\_ be symmetric with respect to exchange of particles. Otherwise, the results would be undefined because permuting the labels would change the energy.

    Not all many-particle interactions work this way. Another common pattern is for the expression to describe an interaction between one central particle and other nearby particles. An example of this is the 3-particle piece of the Stillinger-Weber potential:

    ```
    CustomManyParticleForce* force = new CustomManyParticleForce(3,
        "L*eps*(cos(theta1)+1/3)^2*exp(sigma*gamma/(r12-a*sigma))*exp(sigma*gamma/(r13-a*sigma));"
        "r12 = distance(p1,p2); r13 = distance(p1,p3); theta1 = angle(p3,p1,p2)");
    force->setPermutationMode(CustomManyParticleForce::UniqueCentralParticle);
    ```

    When the permutation mode is set to UniqueCentralParticle, particle p1 is treated as the central particle. For a set of N particles, the expression is evaluated N times, once with each particle as p1. The expression can therefore treat p1 differently from the other particles. Notice that it is still symmetric with respect to p2 and p3, however. There is no guarantee about how those labels will be assigned to particles.

    Distance cutoffs are applied in different ways depending on the permutation mode. In SinglePermutation mode, every particle in the set must be within the cutoff distance of every other particle. If \_any\_ two particles are further apart than the cutoff distance, the interaction is skipped. In UniqueCentralParticle mode, each particle must be within the cutoff distance of the central particle, but not necessarily of all the other particles. The cutoff may therefore exclude a subset of the permutations of a set of particles.

    Another common situation is that some particles are fundamentally different from others, causing the expression to be inherently non-symmetric. An example would be a water model that involves three particles, two of which \_must\_ be hydrogen and one of which \_must\_ be oxygen. Cases like this can be implemented using particle types.

    A particle type is an integer that you specify when you call addParticle(). (If you omit the argument, it defaults to 0.) For the water model, you could specify 0 for all oxygen atoms and 1 for all hydrogen atoms. You can then call setTypeFilter() to specify the list of allowed types for each of the N particles involved in an interaction:

    ```
    set<int> oxygenTypes, hydrogenTypes;
    oxygenTypes.insert(0);
    hydrogenTypes.insert(1);
    force->setTypeFilter(0, oxygenTypes);
    force->setTypeFilter(1, hydrogenTypes);
    force->setTypeFilter(2, hydrogenTypes);
    ```

    This specifies that of the three particles in an interaction, p1 must be oxygen while p2 and p3 must be hydrogen. The energy expression will only be evaluated for triplets of particles that satisfy those requirements. It will still only be evaluated once for each triplet, so it must still be symmetric with respect to p2 and p3.

    Expressions may involve the operators + (add), - (subtract), \* (multiply), / (divide), and ^ (power), and the following functions: sqrt, exp, log, sin, cos, sec, csc, tan, cot, asin, acos, atan, sinh, cosh, tanh, erf, erfc, min, max, abs, floor, ceil, step, delta, select. All trigonometric functions are defined in radians, and log is the natural logarithm. step(x) = 0 if x is less than 0, 1 otherwise. delta(x) = 1 if x is 0, 0 otherwise. select(x,y,z) = z if x = 0, y otherwise. The names of per-particle parameters have the suffix “1”, “2”, etc. appended to them to indicate the values for the multiple interacting particles. For example, if you define a per-particle parameter called “charge”, then the variable “charge2” is the charge of particle p2. As seen above, the expression may also involve intermediate quantities that are defined following the main expression, using ”;” as a separator.

    In addition, you can call addTabulatedFunction() to define a new function based on tabulated values. You specify the function by creating a TabulatedFunction object. That function can then appear in the expression.

    `__init__`(*self*, *particlesPerSet*, *energy*) → CustomManyParticleForce¶
    :   \_\_init\_\_(self, other) -> CustomManyParticleForce

        Create a CustomManyParticleForce.

        |  |  |
        | --- | --- |
        | Parameters: | - **particlesPerSet** (*int*) – the number of particles in each set for which the energy is evaluated - **energy** (*string*) – an algebraic expression giving the interaction energy of each triplet as a function of particle positions, inter-particle distances, angles, and any global and per-particle parameters |

    Methods

    |  |  |
    | --- | --- |
    | `__init__`((self, particlesPerSet, ...) | \_\_init\_\_(self, other) -> CustomManyParticleForce |
    | `addExclusion`((self, particle1, particle2) -> int) | Add a particle pair to the list of interactions that should be excluded. |
    | `addGlobalParameter`((self, name, ...) | Add a new global parameter that the interaction may depend on. |
    | `addParticle`((self, parameters[, type]) | addParticle(self, parameters) -> int |
    | `addPerParticleParameter`((self, name) -> int) | Add a new per-particle parameter that the interaction may depend on. |
    | `addTabulatedFunction`((self, name, ...) | Add a tabulated function that may appear in the energy expression. |
    | `createExclusionsFromBonds`(self, bonds, ...) | Identify exclusions based on the molecular topology. |
    | `getCutoffDistance`((self) -> double) | Get the cutoff distance (in nm) being used for nonbonded interactions. |
    | `getEnergyFunction`((self) -> std::string const &) | Get the algebraic expression that gives the interaction energy of each bond |
    | `getExclusionParticles`(self, index) | Get the particles in a pair whose interaction should be excluded. |
    | `getForceGroup`((self) -> int) | Get the force group this Force belongs to. |
    | `getGlobalParameterDefaultValue`((self, ...) | Get the default value of a global parameter. |
    | `getGlobalParameterName`((self, ...) | Get the name of a global parameter. |
    | `getNonbondedMethod`(...) | Get the method used for handling long range nonbonded interactions. |
    | `getNumExclusions`((self) -> int) | Get the number of particle pairs whose interactions should be excluded. |
    | `getNumGlobalParameters`((self) -> int) | Get the number of global parameters that the interaction depends on. |
    | `getNumParticles`((self) -> int) | Get the number of particles for which force field parameters have been defined. |
    | `getNumParticlesPerSet`((self) -> int) | Get the number of particles in each set for which the energy is evaluated |
    | `getNumPerParticleParameters`((self) -> int) | Get the number of per-particle parameters that the interaction depends on. |
    | `getNumTabulatedFunctions`((self) -> int) | Get the number of tabulated functions that have been defined. |
    | `getParticleParameters`(self, index) | Get the nonbonded force parameters for a particle. |
    | `getPerParticleParameterName`((self, ...) | Get the name of a per-particle parameter. |
    | `getPermutationMode`(...) | Get the mode that selects which permutations of a set of particles to evaluate the interaction for. |
    | `getTabulatedFunction`((self, ...) | getTabulatedFunction(self, index) -> TabulatedFunction |
    | `getTabulatedFunctionName`((self, ...) | Get the name of a tabulated function that may appear in the energy expression. |
    | `getTypeFilter`(self, index) | Get the allowed particle types for one of the particles involved in the interaction. |
    | `setCutoffDistance`(self, distance) | Set the cutoff distance (in nm) being used for nonbonded interactions. |
    | `setEnergyFunction`(self, energy) | Set the algebraic expression that gives the interaction energy of each bond |
    | `setExclusionParticles`(self, index, ...) | Set the particles in a pair whose interaction should be excluded. |
    | `setForceGroup`(self, group) | Set the force group this Force belongs to. |
    | `setGlobalParameterDefaultValue`(self, index, ...) | Set the default value of a global parameter. |
    | `setGlobalParameterName`(self, index, name) | Set the name of a global parameter. |
    | `setNonbondedMethod`(self, method) | Set the method used for handling long range nonbonded interactions. |
    | `setParticleParameters`(self, index, ...) | Set the nonbonded force parameters for a particle. |
    | `setPerParticleParameterName`(self, index, name) | Set the name of a per-particle parameter. |
    | `setPermutationMode`(self, mode) | Set the mode that selects which permutations of a set of particles to evaluate the interaction for. |
    | `setTypeFilter`(self, index, types) | Set the allowed particle types for one of the particles involved in the interaction. |
    | `updateParametersInContext`(self, context) | Update the per-particle parameters in a Context to match those stored in this Force object. |
    | `usesPeriodicBoundaryConditions`((self) -> bool) | Returns whether or not this force makes use of periodic boundary conditions. |

    Attributes

    |  |  |
    | --- | --- |
    | `CutoffNonPeriodic` |  |
    | `CutoffPeriodic` |  |
    | `NoCutoff` |  |
    | `SinglePermutation` |  |
    | `UniqueCentralParticle` |  |

    `getNumParticlesPerSet`(*self*) → int¶
    :   Get the number of particles in each set for which the energy is evaluated

    `getNumParticles`(*self*) → int¶
    :   Get the number of particles for which force field parameters have been defined.

    `getNumExclusions`(*self*) → int¶
    :   Get the number of particle pairs whose interactions should be excluded.

    `getNumPerParticleParameters`(*self*) → int¶
    :   Get the number of per-particle parameters that the interaction depends on.

    `getNumGlobalParameters`(*self*) → int¶
    :   Get the number of global parameters that the interaction depends on.

    `getNumTabulatedFunctions`(*self*) → int¶
    :   Get the number of tabulated functions that have been defined.

    `getEnergyFunction`(*self*) → std::string const &¶
    :   Get the algebraic expression that gives the interaction energy of each bond

    `setEnergyFunction`(*self*, *energy*)¶
    :   Set the algebraic expression that gives the interaction energy of each bond

    `getNonbondedMethod`(*self*) → OpenMM::CustomManyParticleForce::NonbondedMethod¶
    :   Get the method used for handling long range nonbonded interactions.

    `setNonbondedMethod`(*self*, *method*)¶
    :   Set the method used for handling long range nonbonded interactions.

    `getPermutationMode`(*self*) → OpenMM::CustomManyParticleForce::PermutationMode¶
    :   Get the mode that selects which permutations of a set of particles to evaluate the interaction for.

    `setPermutationMode`(*self*, *mode*)¶
    :   Set the mode that selects which permutations of a set of particles to evaluate the interaction for.

    `getCutoffDistance`(*self*) → double¶
    :   Get the cutoff distance (in nm) being used for nonbonded interactions. If the NonbondedMethod in use is NoCutoff, this value will have no effect.

        |  |  |
        | --- | --- |
        | Returns: | the cutoff distance, measured in nm |
        | Return type: | double |

    `setCutoffDistance`(*self*, *distance*)¶
    :   Set the cutoff distance (in nm) being used for nonbonded interactions. If the NonbondedMethod in use is NoCutoff, this value will have no effect.

        |  |  |
        | --- | --- |
        | Parameters: | **distance** (*double*) – the cutoff distance, measured in nm |

    `addPerParticleParameter`(*self*, *name*) → int¶
    :   Add a new per-particle parameter that the interaction may depend on.

        |  |  |
        | --- | --- |
        | Parameters: | **name** (*string*) – the name of the parameter |
        | Returns: | the index of the parameter that was added |
        | Return type: | int |

    `getPerParticleParameterName`(*self*, *index*) → std::string const &¶
    :   Get the name of a per-particle parameter.

        |  |  |
        | --- | --- |
        | Parameters: | **index** (*int*) – the index of the parameter for which to get the name |
        | Returns: | the parameter name |
        | Return type: | string |

    `setPerParticleParameterName`(*self*, *index*, *name*)¶
    :   Set the name of a per-particle parameter.

        |  |  |
        | --- | --- |
        | Parameters: | - **index** (*int*) – the index of the parameter for which to set the name - **name** (*string*) – the name of the parameter |

    `addGlobalParameter`(*self*, *name*, *defaultValue*) → int¶
    :   Add a new global parameter that the interaction may depend on.

        |  |  |
        | --- | --- |
        | Parameters: | - **name** (*string*) – the name of the parameter - **defaultValue** (*double*) – the default value of the parameter |
        | Returns: | the index of the parameter that was added |
        | Return type: | int |

    `getGlobalParameterName`(*self*, *index*) → std::string const &¶
    :   Get the name of a global parameter.

        |  |  |
        | --- | --- |
        | Parameters: | **index** (*int*) – the index of the parameter for which to get the name |
        | Returns: | the parameter name |
        | Return type: | string |

    `setGlobalParameterName`(*self*, *index*, *name*)¶
    :   Set the name of a global parameter.

        |  |  |
        | --- | --- |
        | Parameters: | - **index** (*int*) – the index of the parameter for which to set the name - **name** (*string*) – the name of the parameter |

    `getGlobalParameterDefaultValue`(*self*, *index*) → double¶
    :   Get the default value of a global parameter.

        |  |  |
        | --- | --- |
        | Parameters: | **index** (*int*) – the index of the parameter for which to get the default value |
        | Returns: | the parameter default value |
        | Return type: | double |

    `setGlobalParameterDefaultValue`(*self*, *index*, *defaultValue*)¶
    :   Set the default value of a global parameter.

        |  |  |
        | --- | --- |
        | Parameters: | - **index** (*int*) – the index of the parameter for which to set the default value - **defaultValue** (*double*) – the default value of the parameter |

    `addParticle`(*self*, *parameters*, *type=0*) → int¶
    :   addParticle(self, parameters) -> int
        addParticle(self) -> int

        Add the nonbonded force parameters for a particle. This should be called once for each particle in the System. When it is called for the i’th time, it specifies the parameters for the i’th particle.

        |  |  |
        | --- | --- |
        | Parameters: | - **parameters** (*vector< double >*) – the list of parameters for the new particle - **type** (*int*) – the type of the new particle |
        | Returns: | the index of the particle that was added |
        | Return type: | int |

    `getParticleParameters`(*self*, *index*)¶
    :   Get the nonbonded force parameters for a particle.

        |  |  |
        | --- | --- |
        | Parameters: | **index** (*int*) – the index of the particle for which to get parameters |
        | Returns: | - **parameters** (*vector< double >*) –   the list of parameters for the specified particle - **type** (*int*) –   the type of the specified particle |

    `setParticleParameters`(*self*, *index*, *parameters*, *type*)¶
    :   Set the nonbonded force parameters for a particle.

        |  |  |
        | --- | --- |
        | Parameters: | - **index** (*int*) – the index of the particle for which to set parameters - **parameters** (*vector< double >*) – the list of parameters for the specified particle - **type** (*int*) – the type of the specified particle |

    `addExclusion`(*self*, *particle1*, *particle2*) → int¶
    :   Add a particle pair to the list of interactions that should be excluded.

        In many cases, you can use createExclusionsFromBonds() rather than adding each exclusion explicitly.

        |  |  |
        | --- | --- |
        | Parameters: | - **particle1** (*int*) – the index of the first particle in the pair - **particle2** (*int*) – the index of the second particle in the pair |
        | Returns: | the index of the exclusion that was added |
        | Return type: | int |

    `getExclusionParticles`(*self*, *index*)¶
    :   Get the particles in a pair whose interaction should be excluded.

        |  |  |
        | --- | --- |
        | Parameters: | **index** (*int*) – the index of the exclusion for which to get particle indices |
        | Returns: | - **particle1** (*int*) –   the index of the first particle in the pair - **particle2** (*int*) –   the index of the second particle in the pair |

    `setExclusionParticles`(*self*, *index*, *particle1*, *particle2*)¶
    :   Set the particles in a pair whose interaction should be excluded.

        |  |  |
        | --- | --- |
        | Parameters: | - **index** (*int*) – the index of the exclusion for which to set particle indices - **particle1** (*int*) – the index of the first particle in the pair - **particle2** (*int*) – the index of the second particle in the pair |

    `createExclusionsFromBonds`(*self*, *bonds*, *bondCutoff*)¶
    :   Identify exclusions based on the molecular topology. Particles which are separated by up to a specified number of bonds are added as exclusions.

        |  |  |
        | --- | --- |
        | Parameters: | - **bonds** (*vector< std::pair< int, int > >*) – the set of bonds based on which to construct exclusions. Each element specifies the indices of two particles that are bonded to each other. - **bondCutoff** (*int*) – pairs of particles that are separated by this many bonds or fewer are added to the list of exclusions |

    `getTypeFilter`(*self*, *index*)¶
    :   Get the allowed particle types for one of the particles involved in the interaction. If this an empty set (the default), no filter is applied and all interactions are evaluated regardless of the type of the specified particle.

        |  |  |
        | --- | --- |
        | Parameters: | **index** (*int*) – the index of the particle within the interaction (between 0 and getNumParticlesPerSet()) |
        | Returns: | **types** – the allowed types for the specified particle |
        | Return type: | set< int > |

    `setTypeFilter`(*self*, *index*, *types*)¶
    :   Set the allowed particle types for one of the particles involved in the interaction. If this an empty set (the default), no filter is applied and all interactions are evaluated regardless of the type of the specified particle.

        |  |  |
        | --- | --- |
        | Parameters: | - **index** (*int*) – the index of the particle within the interaction (between 0 and getNumParticlesPerSet()) - **types** (*set< int >*) – the allowed types for the specified particle |

    `addTabulatedFunction`(*self*, *name*, *function*) → int¶
    :   Add a tabulated function that may appear in the energy expression.

        |  |  |
        | --- | --- |
        | Parameters: | - **name** (*string*) – the name of the function as it appears in expressions - **function** (*TabulatedFunction \**) – a TabulatedFunction object defining the function. The TabulatedFunction should have been created on the heap with the “new” operator. The Force takes over ownership of it, and deletes it when the Force itself is deleted. |
        | Returns: | the index of the function that was added |
        | Return type: | int |

    `getTabulatedFunction`(*self*, *index*) → TabulatedFunction¶
    :   getTabulatedFunction(self, index) -> TabulatedFunction

        Get a reference to a tabulated function that may appear in the energy expression.

        |  |  |
        | --- | --- |
        | Parameters: | **index** (*int*) – the index of the function to get |
        | Returns: | the TabulatedFunction object defining the function |
        | Return type: | TabulatedFunction |

    `getTabulatedFunctionName`(*self*, *index*) → std::string const &¶
    :   Get the name of a tabulated function that may appear in the energy expression.

        |  |  |
        | --- | --- |
        | Parameters: | **index** (*int*) – the index of the function to get |
        | Returns: | the name of the function as it appears in expressions |
        | Return type: | string |

    `updateParametersInContext`(*self*, *context*)¶
    :   Update the per-particle parameters in a Context to match those stored in this Force object. This method provides an efficient method to update certain parameters in an existing Context without needing to reinitialize it. Simply call setParticleParameters() to modify this object’s parameters, then call updateParametersInContext() to copy them over to the Context.

        This method has several limitations. The only information it updates is the values of per-particle parameters. All other aspects of the Force (the energy function, nonbonded method, cutoff distance, etc.) are unaffected and can only be changed by reinitializing the Context. Also, this method cannot be used to add new particles, only to change the parameters of existing ones.

    `usesPeriodicBoundaryConditions`(*self*) → bool¶
    :   Returns whether or not this force makes use of periodic boundary conditions.

        |  |  |
        | --- | --- |
        | Returns: | true if force uses PBC and false otherwise |
        | Return type: | bool |

    `__delattr__`¶
    :   x.\_\_delattr\_\_(‘name’) <==> del x.name

    `__format__`()¶
    :   default object formatter

    `__getattribute__`¶
    :   x.\_\_getattribute\_\_(‘name’) <==> x.name

    `__hash__`¶

    `__reduce__`()¶
    :   helper for pickle

    `__reduce_ex__`()¶
    :   helper for pickle

    `__sizeof__`() → int¶
    :   size of object in memory, in bytes

    `__str__`¶

    `getForceGroup`(*self*) → int¶
    :   Get the force group this Force belongs to.

    `setForceGroup`(*self*, *group*)¶
    :   Set the force group this Force belongs to.

        |  |  |
        | --- | --- |
        | Parameters: | **group** (*int*) – the group index. Legal values are between 0 and 31 (inclusive). |

High performance molecular simulation on GPUs


### Navigation

- Application Layer
- Library Layer
  - Core Objects
  - Forces
  - Integrators
  - Extras

©2015, Stanford University and the Authors.
|
Powered by Sphinx 1.3.1
& Alabaster 0.7.7
|
Page source
